# Environmental implications of three *Pleurotus* strain growths for water remediation in the perspective of climate change in New Egyptian Delta

**DOI:** 10.1007/s11356-024-32412-z

**Published:** 2024-02-27

**Authors:** Ahmed E. Ibrahim, Hend Abu Salem, Ahmed Abdelhalim

**Affiliations:** 1 Botany and Microbiology Department, Faculty of Science, Cairo University, Giza, Egypt; 2 Geology Department, Faculty of Science, Cairo University, Giza, Egypt

**Keywords:** Fungal growth parameter, *Pleurorotus* spp., Heavy metals, Water remediation, Unconventional water resources, Environmnetal adaptation, New Egyptian Delta (NED)

## Abstract

Recently, the integrated different interdisciplinary studies derived the environmental solutions of the climate change impacts (e.g., cultivation, wastewater treatment, and managing groundwater resources) (Mesalhy et al. 2020, and Gobashy et al. 2021). Thus, this paper focused on the application of bioremediation to maximize the use of wastewater for new reclamation areas in the Northwest Egyptian desert (New Egyptian Delta (NED). In the NED project, the drainage water samples collected from Nile Delta drains will provide the main unconventional water resources for irrigation through the new Hammam canal. Therefore, three *Pleurotus* strains were grown moderately on two natural media, the first containing *Salvia* L. (sage) extract (MDA) and the second containing *Thymus vulgaris* L. (origanum thymus Kuntze, *Thymus collinus* Salisb) (TDA) extract replacing potato infusions in standard PDA. *Pleurotus ostreatus* (Jacquin; Kummer) strain records the highest growth among the three tested fungi on modified media. PO records 4.49 and 4.41 cm on (MDA) and (TDA), respectively. There is a marked decrease in the majority of heavy metal concentrations on sterile drainage water amended with PD broth and inoculated with three tested *Pleurotus* strains individually. At the end of the incubation period, *Pleurotus ostereatus* which expressed in abbreviation (PO) are more efficient in the removal of Al, Co, Cr, and Ni by 53.15, 95.87, 58.47, and 85.07%; respectively. *Pleurorotus pulmonarius* (Fr.) which symbolized (PP) is more potent in the removal of Cd, Si, Sn, Sr, and V by 70.37, 56.59, 41.19, 52.78, and 96.24%; respectively. *Pleurotus floridanus* (NZOR) which indicated as (PF) is actively over the former species in the removal of Ba, Fe, and Mo by 87.84, 46.67, and 97.34%; respectively. Cu, Mn, Pb, As, and Se could not be detected as the control sample recorded measurements below 0.009 mg L^−1^. An unexpected increase in Zn among the different treatments was detected from 05.04 to 07.01%.

## Introduction

**Fig. 1:**
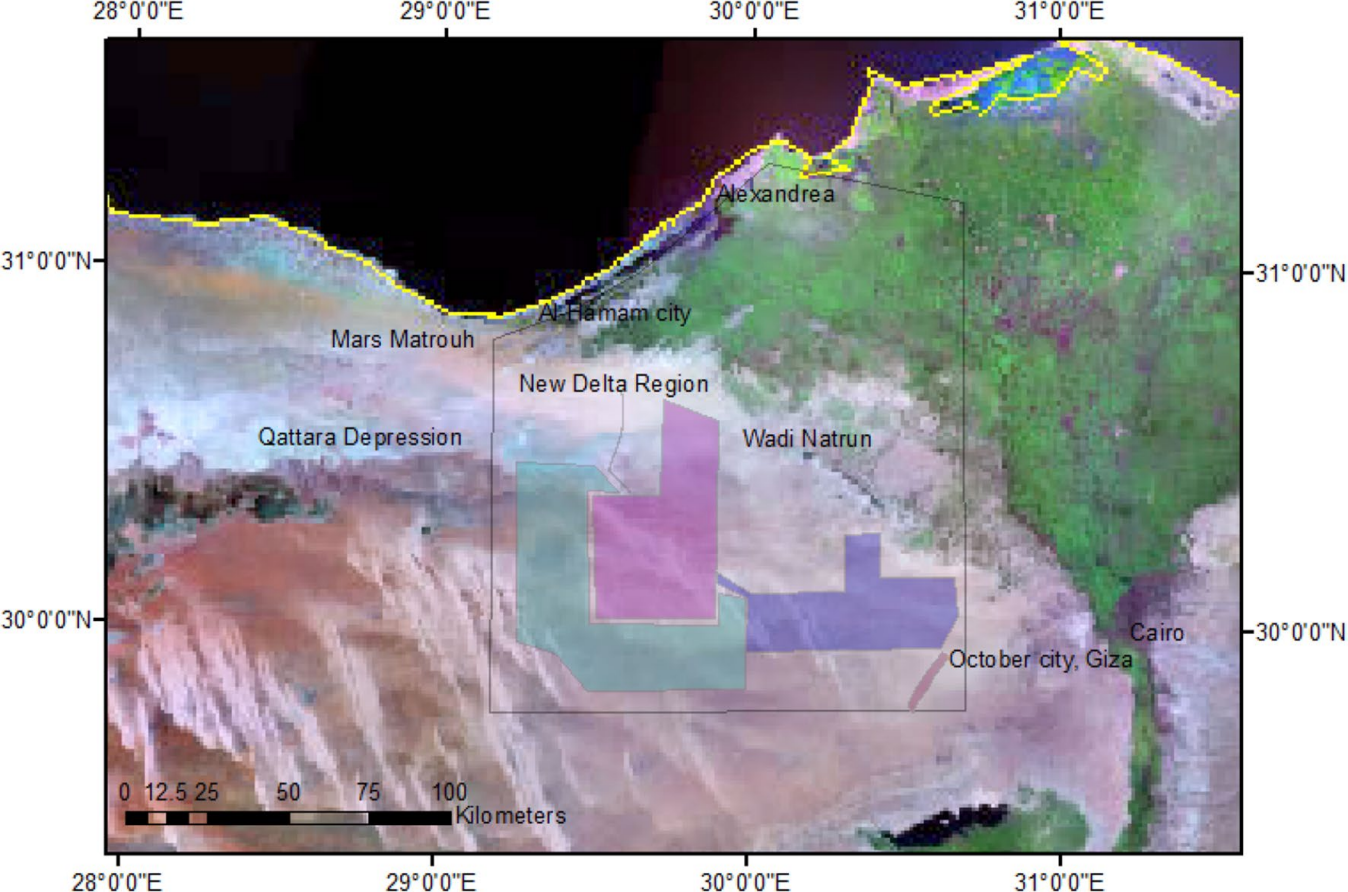
The location map of the new Egyptian delta (NED) three zones based on satellite image

To combat global warming, several methods were used to reduce greenhouse gas emissions (e.g., cultivation, use of clean energy, and CO_2_ sequestration). The widely used method is the cultivation to provide carbon sinks to help attain net zero targets. The challenge needed for cultivation is the presence of water resources in the proposed areas. In Egypt, the New Egyptian Delta (NED) is well known by its arid to semi-arid climate where the water resource is mainly groundwater. Accordingly, to develop this area, the Egyptian government planned to construct El Hammam canal that collects agricultural drainage from the Nile Delta drains reaching El Hammam city (in Matrouh Governorate to the north of the study area) and then moving upslope to the south where the NED is located to irrigate 362,000 feddans after treatment in three project phases (Fig. 1).

*Pleurotus* spp. (oyster mushroom) is one member of the family Tricholomataceae and frequently grows naturally in clusters on dead tree debris during spring (Elattar 2019). Among all mushrooms, oyster mushrooms are the second most widely cultivated types all over the world, after *Agaricus bisporus* (Kües and Liu 2000).

*Pleurotus* species are common and broadly cultivated everywhere in the world due to their availability, simple production technology, low cost, and great biological efficiency (Mane et al. 2007). *Pleurotus* species are effective microorganisms in lignin degradation and can be grown on a wide variety of agricultural wastes within a broad range of temperatures (Jandaik and Goyal 1995). They have high economic, environmental, and medicinal importance. Moreover, they can colonize and degrade many substrates of lignocellulosic materials and various wastes that are produced in forestry, agricultural, and food industries **(**Sánchez 2010).

The main dietary sources of *Pleurotus* oyster mushroom species are hemicellulose, cellulose, and lignin, and most of these substrate materials require nitrogen source amendments such as rice bran and wheat to attain the proper C/N ratio (Siddhant et al. 2013). A variety of industrial and agricultural wastes are used to grow *Pleurotus* species such as baby leaf lettuce (Hernández et al. 2021), sawdust mulch (Girmay et al. 2016), cardboard, and coffee industry waste (Gąsecka et al. 2020). The use of agricultural residues in biological processes may be one solution for the bioconversion of inedible macromolecule residues into food rich in proteinase biomass represented by edible mushrooms (Mshandete et al. 2008 and Fawzi 2016).

Heavy metals are permanent pollutants in the ecosystem as they cannot be removed. They are natural deposits in the earth’s crust, mostly in soil, rock, and water with considerable concentrations that cause environmental pollutions (Mohammed et al. 2011). Most heavy metals are commonly known to be toxic and carcinogenic where they can be discharged into the natural environment through natural processes as well as human activities. Accordingly, ecological changes in nature due to acidification, weathering, and erosion represent the main causes that bring heavy metals to the environment (Tajam and Kamal 2013; Paul 2017). For example, heavy precipitation or run-off can cause heavy metals to leak out of geological formations. Occasionally, anthropogenic activities such as agricultural operations, household waste, industrial processes, landfills, and sewage disposal feedback heavy metals again to the environment (Mudhoo et al. 2012; and Elgarahy et al. 2021).

Some fungi can remove dissolved metals from water in the environment (Shivakumar et al. 2011; Mishra and Malik 2012). Compared with physical and chemical techniques, which are very expensive and energy consuming and may cause secondary environmental pollution (Price et al. 2001), the use of bio-assimilative fungi is very useful and promising for decontaminating wastewater from persistent organic pollutants (POPs)/heavy metals (Chen et al. 2011). However, in contrast to the decomposition mechanisms of POPs, the basis of the heavy metal removal process by fungi is commonly very limited. In general, these fungi have been found to use two heavy metal accumulation methods, namely intracellular bioaccumulation (Mishra and Malik 2012) and surfactant extracellular adsorption (Xu et al. 2014). Fungi can absorb heavy metals and bio-accumulate them intracellularly.

*Pleurotus eryngii* cultivation on natural substrate amended with thymus (*Thymus vulgaris*) post‐extraction waste (TPEW) can beneficially enhance the net-yield production depending on their elevation of Ca and Na contents and it considered as much low costive substrate than the other ones (Gąsecka et al. 2020). *Salvia miltiorrhiza* also controls the infection on edible mushrooms by *Pseudomonas stutzeri* and *Pseudomonas tolaasii* as common pathogenic bacteria that delimit mushroom production. The phytochemical management of mushroom pathogens is safer than chemicals (bactericides) due to their higher toxicity during human mushroom consumption (Dawoud and Eweis 2006). In our current work, we use alternative natural source to potatoes in synthetic media (*Thymaus vulgaris* L. *and Slavia officinalis* L.) to support yield, to be economically costive, and also protect edible mushroom against bacterial pathogens during cultivation.

To maximize the benefit of using untreated wastewater, three isolates of wild mushroom belonging to *Pleurotus* spp. were used to improve the properties of irrigation water by minimizing the load of heavy metals as well as organic compounds in wastewater to be efficient in irrigation.

## Material and methods

### Strain collection

Three tested strains of *Pleurotu*s spp. were taken in the form of commercial fruiting body packages from Agriculture Research Center, Giza, Egypt. The three isolates were *Pleurotus ostreatus* (PO), *Pleurotus pulmonarius* (PP), and *Pleurotus floridanus* (PF). The fresh fruiting bodies of each isolate were cut into small pieces; surface sterilization was carried in 5% sodium hypochlorite for 120 s and rinsed three times in sterile bi-distilled water. Mushroom pieces were then cultivated on PDA in 3-cm Petri dishes and incubated at 25 °C for 7 days. After the incubation period, fungal growth was examined; mycelial extensions were purified and kept in slants at 5–10 °C till use (Ibrahim 2005 and Sharma, et al. 2013).

### Experimental planning

Three experiments were designed. The first one was performed to evaluate the ability of three isolates of *Pleurotus* spp. to grow on natural plant extract agar media compared with standard potato dextrose agar (PDA) media; the second and third experiments were performed to determine the potentiality of the tested strains for the remediation of wastewater from dyes and heavy metals. All experiments were carried out in the Botany and Microbiology and in the Geology Departments, Faculty of Science, Cairo University. All treatments were carried out in triplicates for each treatment. Each experiment was conducted three times, and the represented data recovered from the mean of values were recorded from these experiments.

### Natural media used in the growth of different *Pleurotus* isolates

The following culture media were used:

#### Potato dextrose agar (PDA)

PDA growth medium was prepared as follows: 200 g infusion of potato tubers, 20.0 g D (+) dextrose, 15 agar, and 1 L distilled water, and then autoclaved at 121 °C for 20 min (Beever and Bollard 1970).

#### Marmaria dextrose agar (MDA)

MDA medium was prepared by adding the extract of 20 g from dried plant debris of *Salvia officinalis* L. (sage), 20.0 g D (+) dextrose, 15 agar, and 1 L distilled water, filtered and then autoclaved, as described earlier.

#### Thyme dextrose agar (TDA)

TDA medium was prepared by adding 20 g infusion from dried plant debris of *Thymus vulgaris* L. (*Origanum thymus* Kuntze, *Thymus collinus* Salisb), 20.0 g D (+) dextrose, 15 agar, and 1 L distilled water, filtered, and then autoclaved, as mentioned before.

Additionally, the pH of each of the above media was adjusted at 6.5 and then poured in sterilized 9-cm Petri dishes. Streptomycin was amended after sterilization at the rate of 0.1 g/100 mL medium. The medium was cooled till 40 °C; and then the strains were inoculated by (0.5 mm^2^) of inverted inoculum from tested *Pleurotus* (The final step will be repeated with three isolates using 0.5-mm^2^-sterile cork borer for each isolate). The above inoculate media were incubated for 10 days at 25 °C. The radial growth was calculated according to the method reported by Zharare et al. (2010). The growth rate was derived from the following formula (Nguyen and Ranamukhaarachchi 2020):

$$\mathrm{Growth\;rate }=\frac{\mathrm{colony\;diameter\;on\;the\;last\;day\;}({\text{cm}})}{\mathrm{number\;of\;days}}$$

The above steps were repeated by using potato dextrose broth (PD), Marmaria dextrose broth (MD), and thyme dextrose broth (TM). The inoculated liquid cultures were then incubated at 25 °C for 10 days. After the incubation period, the fresh and dry weights of fungal discs were measured and tabulated (Nguyen and Ranamukhaarachchi 2020).

$$\mathrm{Growth\;rate }=\frac{\mathrm{dry\;weights\;on\;the\;last\;day}({\text{cm}})}{\mathrm{number\;of\;days}}$$

### Drainage water amended to PDA growth media, then inoculated with *Pleurotus* spp.

Three *Pleurotus* strains were allowed to grow in the solid PDA media at 25 °C and pH 6.5 and then incubated for 7 days. An agar plug (5 mm in diameter) of each strain was cut from the margin of the media and then used to inoculate 250-mL-conical flasks containing 100 mL of sterile drainage water amended to the liquid PD broth (1:1 v/v). Each treatment was conducted in triplicate. After 14 days of incubation at 25 °C, the liquid cultures were filtered (The method was carried out according to Yang et al. 2017 with some modifications). The concentrations of heavy metals were estimated in un-inoculated media as a control and also measured for the corresponding media inoculated with each *Pleurotus* strains (PO, PP, and PF) separately. The experiment was also repeated on solid media (PDA) with the same ingredients and sub-culturing of different isolates performed in 3-cm plates (in triplicates) and then, after 14 days of incubation (25 °C), the mycelia growths for different isolates were examined microscopically.

The percentage of inhibition or activation is derived from the following formula:

$$\frac{\mathrm{The\;concentration\;of\;element\;in\;control }-\mathrm{the\;concentration\;of\;element\;in\;treatment}}{\mathrm{the\;concentration\;of\;element\;in\;control}}\times\;100\%$$

The positive values referred to inhibition, and the negative expressed activation of elements.

The drainage water used for irrigation was collected from agricultural drainage that will feed El Hammam Canal in the NED. The drainage samples were mixed well and amended to the fungal growth media in a selected ratio during the current assay.

The major ions’ chemistry of the drainage water was analyzed in the central laboratories of the National Water Research Centre, Qanater Al Khairiya, Egypt, according to APHA (2017) using Ion Chromatography model Dionex ICS5000. The trace and heavy elements such as Al, B, As, Sb, Cd, Co, Cr, Cu, Fe, Mo, Mn, Ni, Pb, Sr, V, and Zn were detected by inductively coupled plasma-optical emission spectrometry (ICP-OES), Thermo Jarrell Elemental Company, USA) at the central laboratories of the Desert Research Center.

### Water quality assessment for irrigation

The suitability of the studied water samples for irrigation is assessed using Kelly’s ratio (*KR*), sodium percent (Na%), sodium adsorption ratio (*SAR*), residual sodium carbonate (*RSC*), magnesium ratio (*MR*), and permeability index (*PI*). Additionally, US Salinity Laboratory staff (USSL Staff 1954) diagram is used to classify water based on the dual effect of salinity hazard (expressed by EC) and sodium hazard (expressed by SAR). Wilcox (1948) diagram is also used to study the suitability of water for irrigation.

### Statistical analysis

The results were conducted in a complete randomized system with 3 replicates; therefore, the data were analyzed and oriented to analysis of variance, and the means of individuals were compared through modified Duncan’s multiple range test (Berry and Hochberg 1999) using SPSS 16. The difference in letters (a, b, and c) within the same isolate on different media or between different isolates on the same medium were considered of significance.

## Results

### Experiment 1(a). Measuring of growth parameters (radial growth and growth rates) of the different *Pleurotus* spp. on different tested natural media.

In this experiment, in fusion of 20 g of either *Meramiya* or *Thymus* was selected from preliminary test associated with optimum growth in the two plant infusions among all tested *Pluerotus* strains compared to standard PDA.

**Fig. 2:**
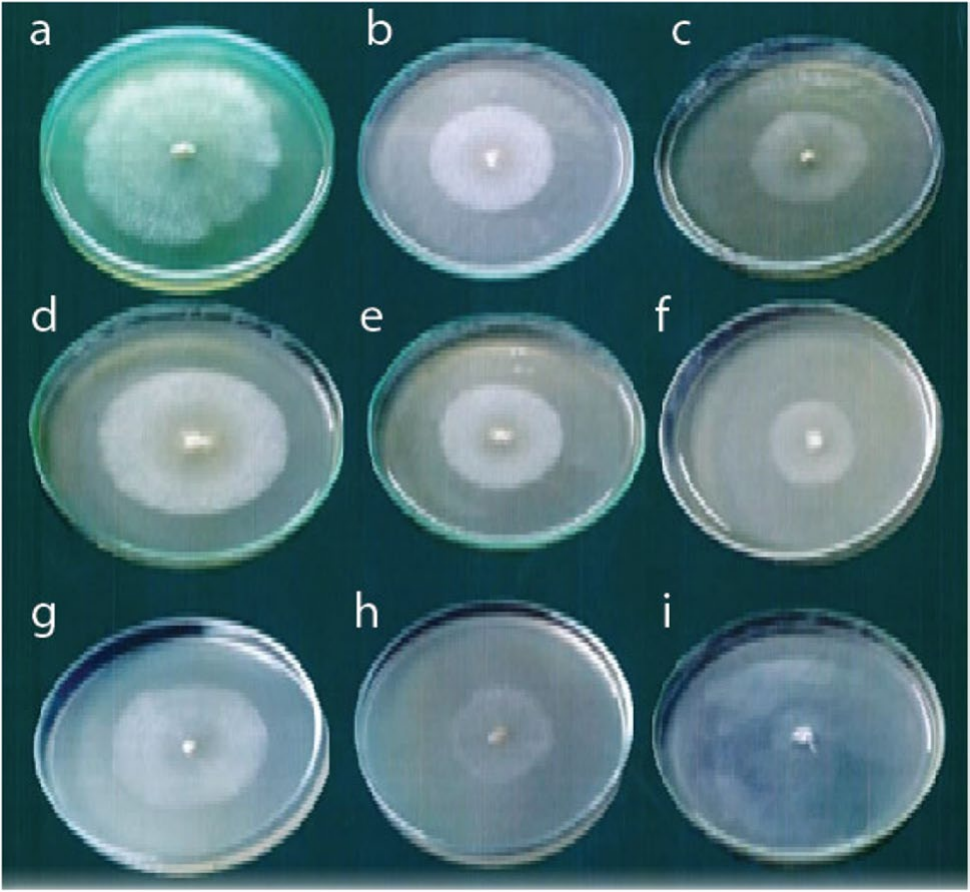
Radial growth of different *Pleurotus* spp. on different *Pleurotus*-tested media: row one: radial growth of P. O. on **a** PDA, **b** MDA, **c** TDA for 7 days at 25 °C. Row two: radial growth of P. F. on **d** PDA, **e** MDA, and **f** TDA for 7 days at 25 °C. Row three: radial growth of PP on **g** PDA, **h** MDA, and **i** TDA for 7 days at 25 °C

Therefore, three natural agar media (PDA, MDA, and TDA) were used to examine the radial growth and growth rates for the three tested isolates of *Pleurotus* spp. Data presented in Table 1 and Fig. 2 reveal that the gradual decrease of the estimated radial growth on a standard PDA measures 6.41, 5.93, and 5.21 cm with PO, PF, and PP, respectively. The gradual significant drop of the measured radial growth on an MDA records 4.49, 3.86, and 3.25 cm with PO, PF, and PP, respectively. Additionally, the significant decrease of the estimated radial growth on TDA measures 4.43, 3.31, and 2.12 with PO, PF, and PP, respectively. The data also showed a significant decrease in growth parameters regarding PF and PP compared with PO on different media.

**Table 1:** Effect of different media on radial growth (cm) of different *Pleurotus* spp. grown on different media for 7 days at 25 °C and pH = 6.5

Growth parameter	Mycelial diameter (cm) and growth rate (cm/day) after 1 week incubation period						L.S.D. (*P* ‹ 0. 5)	
Media	PO		PP		PF			
	D.W	G.R	D.W	G.R	D.W	G.R	D.W	G.R
PDA	6.41 ± 0.39^aa^	0.90 ± 0.07^aa^	5.21 ± 0.28^ac^	0.79 ± 0.03^ac^	5.93 ± 0.26^ab^	0.84 ± 0.05^ab^	0.81	0.039
MDA	4.49 ± 0.54^ba^	0.62 ± 0.09^ba^	3.25 ± 0. 32^bc^	0.53 ± 0.08^bc^	3.86 ± 0.21^bb^	0.58 ± 0.05^bb^	0.74	0.043
TDA	4.43 ± 0.27^ba^	0.59 ± 0.04^ba^	1.92 ± 0.62^bc^	0.51 ± 0.08^bc^	3.31 ± 0.37^bb^	0.55 ± 0.08^bb^	0.68	0.035
L.S.D. (*P* ‹ 0.5)	0.92	0.025	0.63	0. 019	0.59	0.025		

Means characterized by identical letters referred insignificantly different values (*P* ‹ 0.5) according to modified Duncan’s multiple range test and standard deviation. Significance of organism on different media expressed by the 1st letter and for significance between different organisms on the same media, expressed by the 2nd letter

*D*.*W.* means the diameter for radial growth measurement (cm), *G.R.* means the growth rate

### Experiment 1(b). Measuring of growth parameters (dry weight and growth rates) of the different *Pleurotus* spp. on different tested natural broth media.

In this experiment, three liquid media (PD broth, TD broth, and MD broth) were used to examine the dry weights and growth rates for the three tested strains of *Pleurotus* spp. Data presented in Table 2 show gradual decrease of the estimated dry weights on a standard PDA broth measures 2.98, 2.78, and 2.32 cm with PO, PF, and PP; respectively. The gradual drop of the measured radial growth on the MDA records 2.32, 1.96, and 1.53 mg, with PO, PF, and PP; respectively. The gradual decrease of the estimated radial growth on a standard TDA measures 1.92, 1.62, and 1.41 mg with PO, PF, and PP; respectively. The data also show a significant decrease in growth parameters regarding PF and PP compared with PO on different media.

**Table 2:** Effect of different media on dry weight (mg/100 mL medium) of different *Pleurotus* spp. grown on different media for 7 days at 25 °C and pH = 6.5

Growth parameter	Mycelial dry weights (mg) and growth rate mg/day after 1 week incubation period						L.S.D. (*P*‹ 0.5)	
Media	PO		PP		PF			
	D.W	G.R	D.W	G.R	D.W	G.R	D.W	G.R
PDA	2.98 ± 0.25^aa^	0.87 ± 0.05^aa^	2.32 ± 0.36^ac^	0.79 ± 0.09^ac^	2.78 ± 0.65^ab^	0.73 ± 0.08^ab^	0.53	0.047
MDA	2. 32 ± 0.62^ba^	0.58 ± 0.08^ba^	1.53 ± 0.52^bc^	0.48 ± 0.02^bc^	1.96 ± 0.34^bb^	0.54 ± 0.02^bb^	0.67	0.037
TDA	2. 12 ± 0.41^ba^	0.55 ± 0.02^ba^	1.41 ± 0. 29^bc^	0.43 ± 0.05^bc^	1.62 ± 0.29^bb^	0.49 ± 0.06^bb^	0.59	0.041
L.S.D. (* P*‹ 0.5)	0.35	0.027	0.49	0.029	0.46	0.018		

Means characterized by identical letters referred insignificantly different values (*P*‹ 0.5) according to modified Duncan’s multiple range test; standard deviation for one organism on different media, 1st letter and between different organisms on the same media, 2nd letter

*D.W.* means the diameter for radial growth measurement (cm). *G.R.* means the growth rate

### Experiment 2. Efficiency of *Pleurotus* isolates in remediation of heavy metals. (1:1 v/v)

In this experiment, drainage water amended to the PD broth (1:1 v/v) and the used ratio of drainage water/PD for their optimum fungal growth among different ratios of drainage water/PD mixtures. The selected ratio chosen after preliminary tests for average (65–70%) growth recorded by tested isolates was compared with standard PD broth. The growth of all isolates recorded fresh weights, 9.478 to 7.301 mg/100 mL medium, and dry weights were measured from 1.508 to 1.624 mg/mL compared with zero growth (drainage water without any amendments). The average growth for all isolates on standard PD broth was measured (fresh weights 14, 53 to 14.97 mg/100 mL medium, and dry weights were measured 2.32 to 2.98 mg/mL).

Data in Table 3 show that a marked reduction in most estimated heavy metals in inoculated natural media (drainage water + PD broth) with three tested *Pluerotus* spp. Al, Ba, Cd, Co, Fe, Cr, Mo, Ni, Si, Sn, Sr, and V are much decreased with all inoculated tested *Pluerotus* spp.

**Table 3:** Potentiality of the isolated fungi to remove heavy metals in sterile drainage water amended to the liquid PD broth (1:1 v/v) after incubation for 14 days at 25 °C, pH = 6.5

Treatments	Heavy metal in control as in fungal inoculated media measured in mg L^−1^						
	Control	PO		PP		PF	
Heavy metals	Conc	Conc	% of inhibition or activation	Conc	% of inhibition or activation	Conc	% of inhibition or activation
Al	0.2286	0.1071	53.15%	0.2031	11.15%	0.1866	18.37%
Ba	1.2060	0.1638	86.42%	0.172	85.74%	0.1467	87.84%
Cd	0.0432	0.0276	36.11%	0.0128	70.37%	0.0297	31.25%
Co	0.0242	‹ 0.001	95.87%	‹ 0.001	95.87%	‹ 0.001	95.87%
Cr	0.2798	0.1162	58.47%	0.1499	46.42%	0.1771	36.70%
Cu (U)	‹ 0.006	‹ 0.006	Not detected	‹ 0.006	Not detected	‹ 0.006	Not detected
Fe	0.3441	0.2648	23.04%	0.3112	9.56%	0.1835	46.67%
Mn (U)	‹ 0.002	‹ 0.002	Not detected	‹ 0.002	Not detected	‹ 0.002	Not detected
Mo	0.0376	0.0099	73.67%	0.0216	42.55%	‹ 0.001	97.34%
Ni	0.0134	‹ 0.002	85.07%	‹ 0.002	85.07%	0.0116	13.43%
Pb (U)	‹ 0.008	‹ 0.008	Not detected	‹ 0.008	Not detected	‹ 0.008	Not detected
Si	16.74	12.54	25.09%	7.266	56.59%	11.07	33.87%
Sn	1.155	1.127	02.42%	0.6793	41.19%	0.8944	22.56%
Sr	3.592	2.786	22.44%	1.696	52.78%	2.578	28.23%
V	0.2664	0.2474	07.13%	‹ 0.01	96.24%	0.0337	87.35%
Zn (A)	0.0405	0.0689	07.01%	0.0609	05.04%	0.0656	06.30%
As (U)	‹ 0.001	‹ 0.001	Not detected	‹ 0.001	Not detected	‹ 0.001	Not detected
Se (U)	‹ 0.002	‹ 0.002	Not detected	‹ 0.002	Not detected	‹ 0.002	Not detected

(A) means that the element is activated by all tested strains. (U) means the elements show undetectable variation among all treatments including control sample

**Fig. 3:**
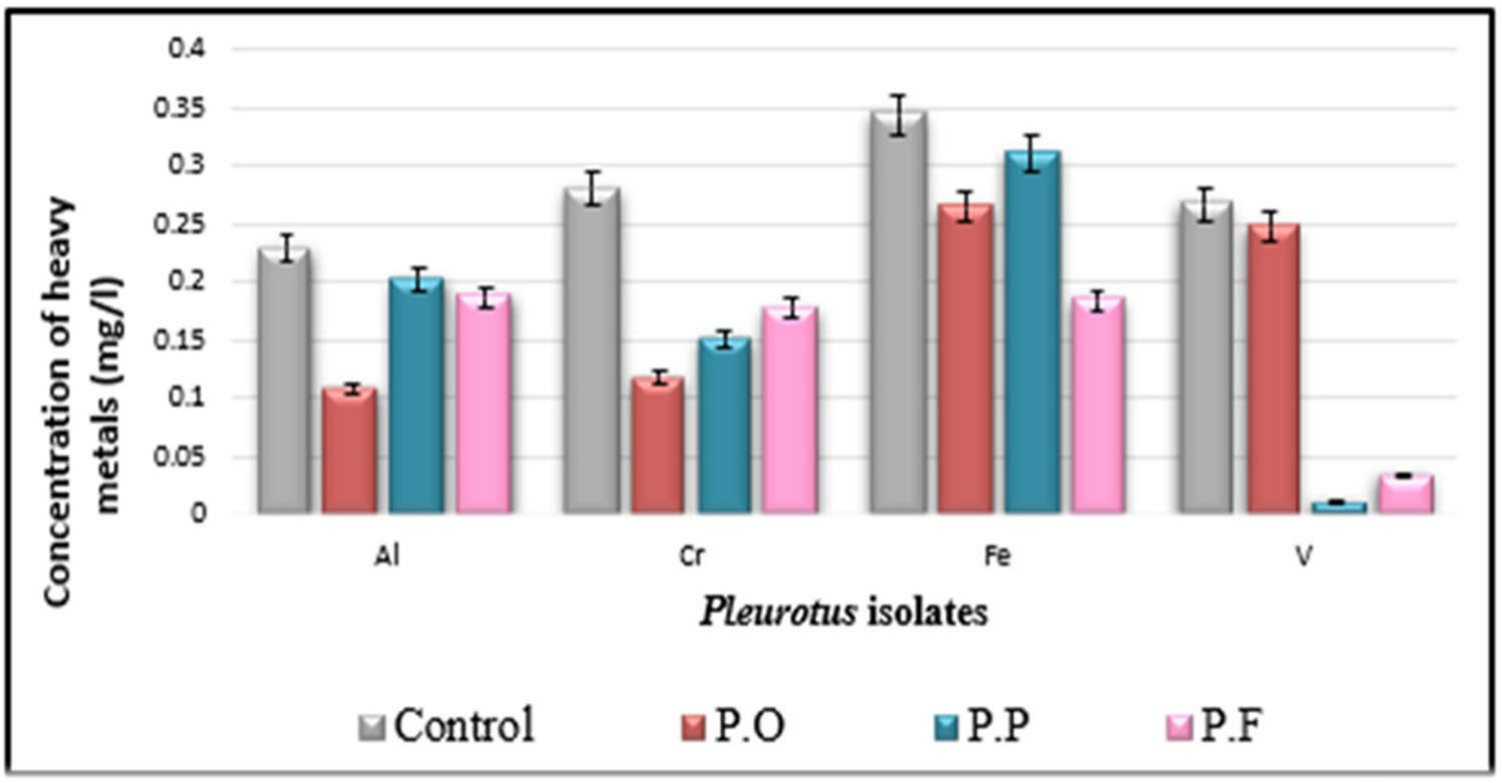
Water bioremediation for Al, Cr, Fe, and V by different *Pleurotus* isolates

**Fig. 4:**
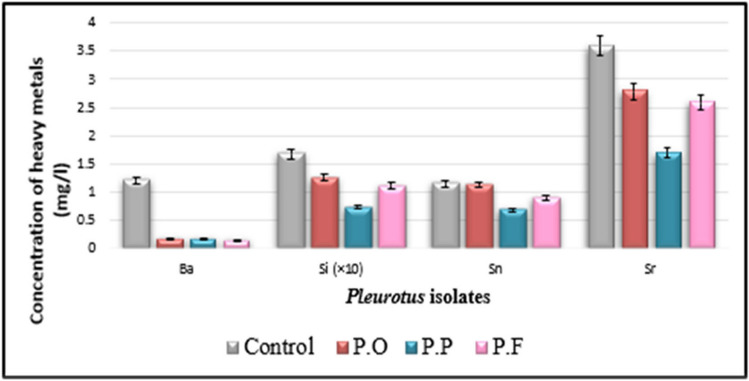
Water bioremediation for Ba Si, Sn, and Sr by different *Pleurotus* isolates

**Fig. 5:**
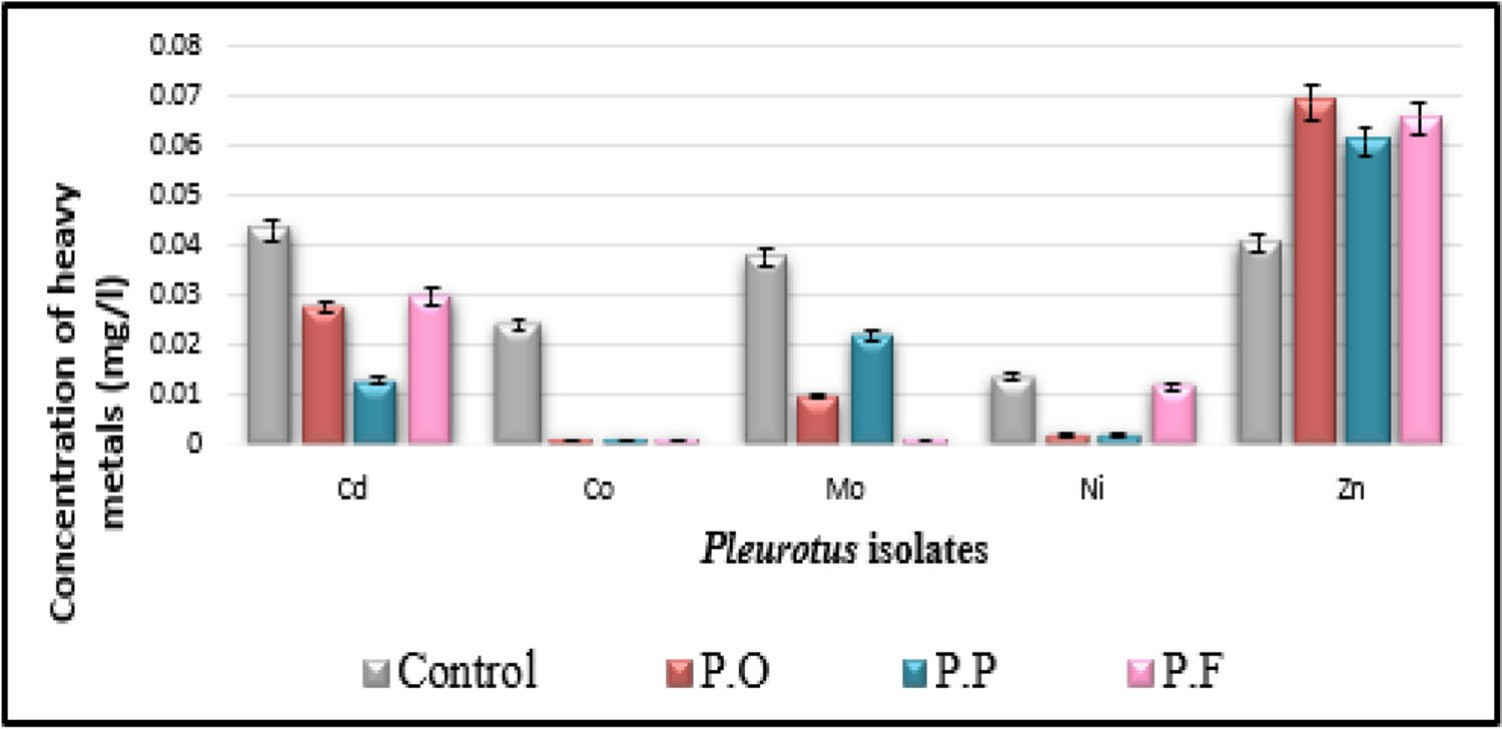
Water bioremediation for Cd, Co, Mo, Ni, and Zn by different *Pleurotus* isolates

**Fig. 6:**
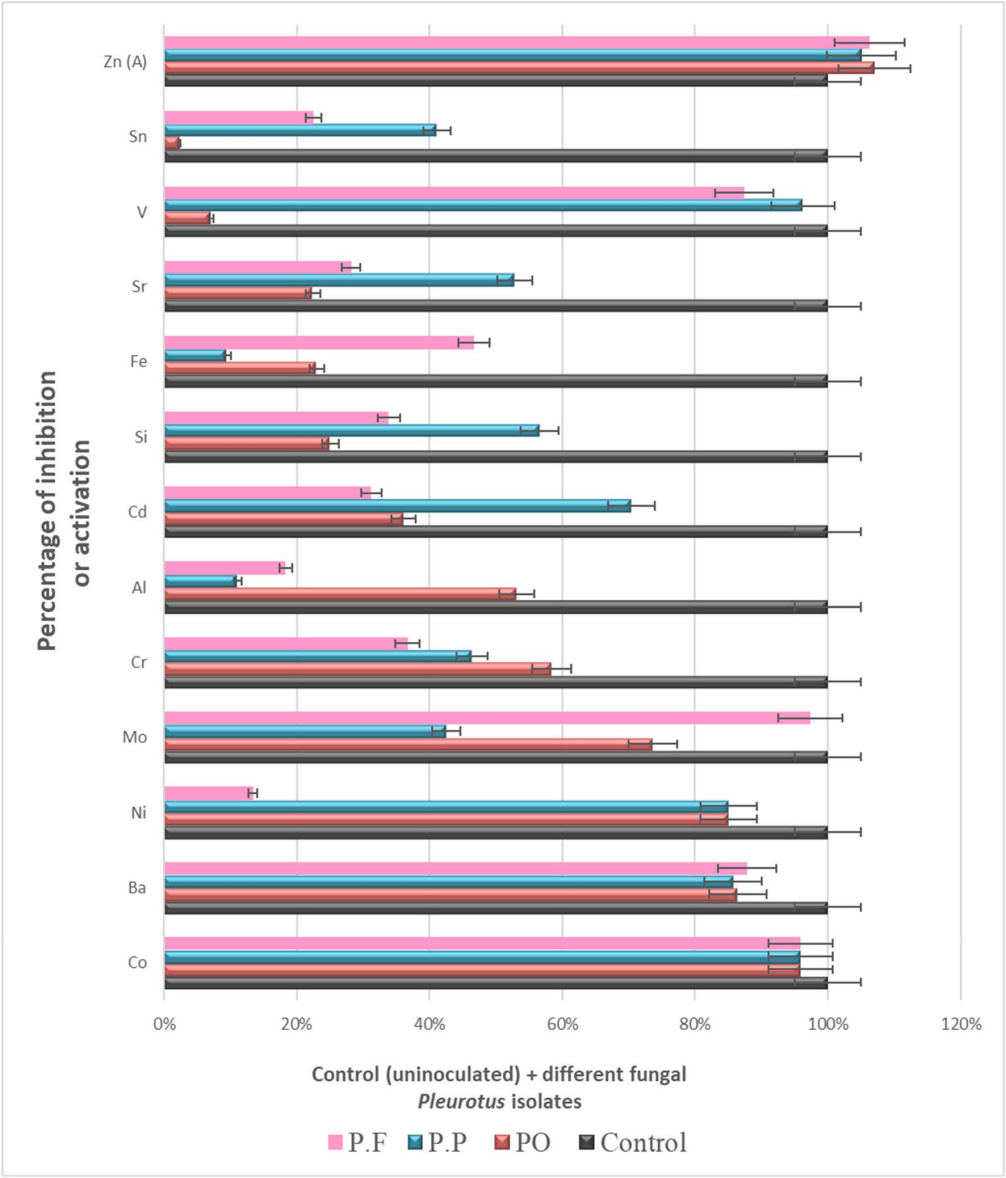
Percentage of inhibition or activation of heavy metals regarding to the efficiency of the different *Pleurotus* spp. compared with un-inoculated control

The higher efficiency of removal of heavy metals was recorded with the following treatments (Figs. 3, 4, 5, and 6); Al decreased from 0.2286 to 0.1071 mg L^−1^ with PO isolate, 53.15% of reduction; Ba decreased from 1.2060 to 0.1467 mg L^−1^ with PF isolate, 87.84% of reduction; Cd decreased from 0.0432 to 0.0128 mg L^−1^ with PP isolate, 70.37% of reduction; Co decreased from 0.0242 to lower than 0.001 mg L^−1^ with all fungal strains, 95.87% of reduction; and Cr decreased from 0.2798 to 0.1162 mg L^−1^ with PO strain, 58.47% of reduction. Fe decreased from 0.3441 to 0.1835 mg L^−1^ with PF strain, 46.67% of reduction, Mo decreased from 0.0376 to lower than 0.001 mg L^−1^ with PF strain, 97.34% of reduction; Ni decreased from 0.0134 to lower than 0.002 mg L^−1^ with both PO and PP strain, 85.07% of reduction; Si decreased from 16.74 to 7.266 mg L^−1^ with PP strain, 56.59% of reduction; Sn decreased from 1.155 to 0.6793 mg L^−1^ with PP strain, 41.19% of reduction; Sr decreased from 3.592 to 1.696 mg L^−1^ with PP strain, 52.78% of reduction; and V decreased from 0.2664 to lower than 0.01 mg L^−1^ with PP strain, 96.24% of reduction.

Some elements are undetectable where the measured values were less than 0.009 mg L^−1^. All fungal strains exhibit a slight increase in Zn from 05.04 to 07.01% compared with control which measured 0.0405 mg L^−1^.

### Water quality assessment

In order to study the suitability of the studied water samples (raw agricultural wastewater and three samples extracted after experiments) for irrigation purposes, we calculated several parameters that are discussed below.

- 1. Kelly’s ratio

*KR* is one of the parameters used to evaluate water suitability for irrigation. It is calculated by the following equation:

$$KR=\frac{{{\text{Na}}}^{+}}{{{\text{Ca}}}^{2+}+{{\text{Mg}}}^{2+}}\;{\text{meq}}/{\text{L}}.$$  

**Fig. 7:**
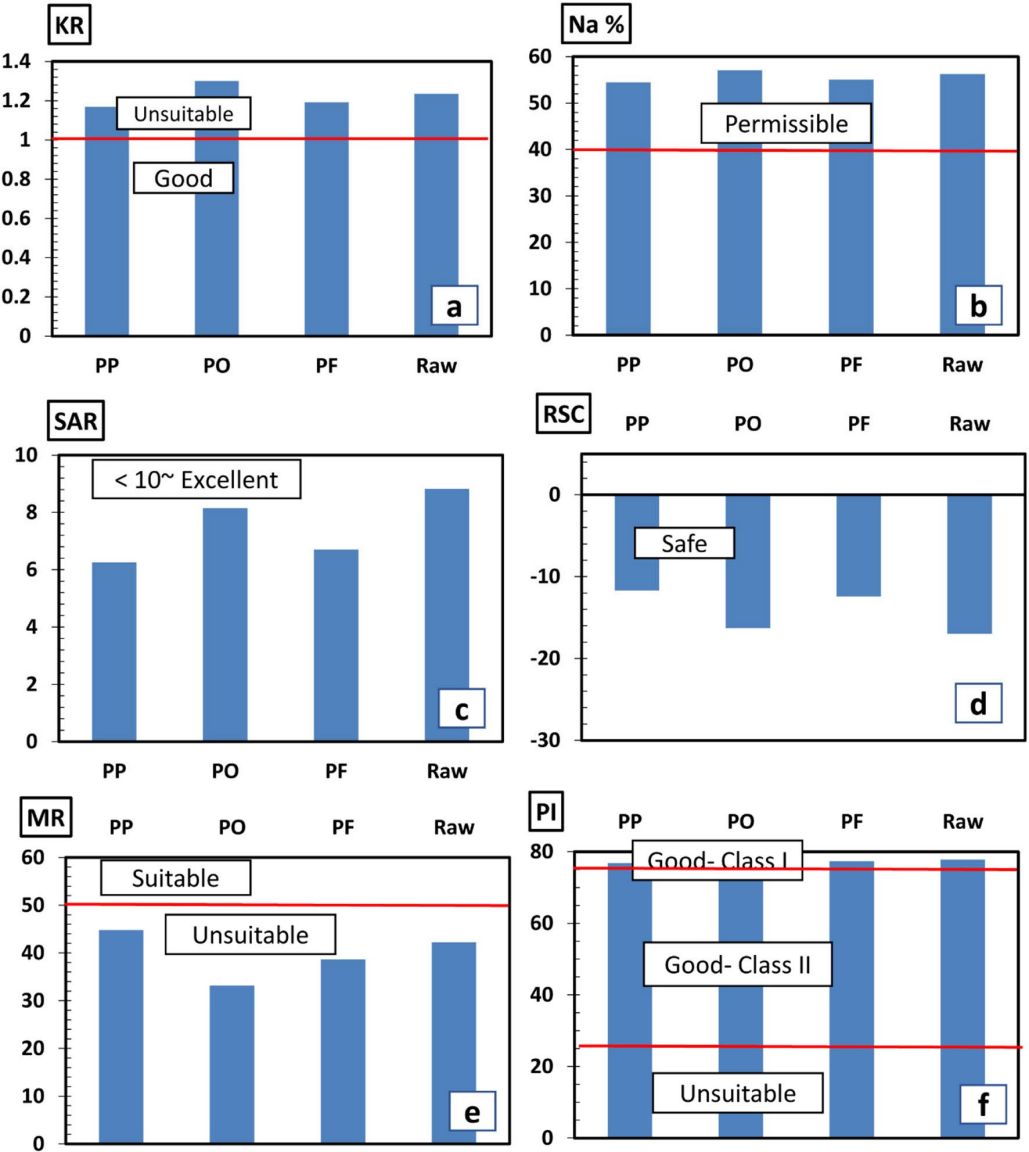
Distribution charts of the studied water samples’ suitability for agricultural uses based on, **a** Kelly’s ratio, **b** Na%, **c** *SAR*, **d** *RSC*, **e** *MR*, and **f** *PI*

A ratio equals to or less than 1 indicates a good quality of water for irrigation, while if it is more than 1, this means the unsuitability of water for irrigation due to high sodium content (USSL Staff 1954; Al-Ruwaih et al. 2019 and Abu Salem et al. 2023). All the studied samples have *KR* of more than 1 (Fig. 7a).

- 2. Sodium percent

Na% is used to evaluate the water quality for agricultural purposes (Wilcox 1948). It is calculated by the following equation:

$$\mathrm{Na\%} = (({{\text{Na}}}^{+}+{{\text{K}}}^{+})/({{\text{Ca}}}^{2+}+{{\text{Mg}}}^{2+}+{{\text{Na}}}^{+}+{{\text{K}}}^{+}))\times\;100\;{\text{meq}}/{\text{L}}.$$  

Water quality classes based on the Na% are given in Table 4, where the acceptable sodium content in irrigation water usually ranges from 0 to 40 meq/L (Ayers & Westcot 1985). The increased sodium percent in irrigation water poses considerable hazards to plant growth as well as soil permeability reduction (Joshi et al. 2009). The studied samples have Na% from 54.4 to 57.06% (Fig. 7b).

**Table 4:** Parameters’ ratings of water quality for irrigation purposes

Parameter	Range	Water quality
*KR*	Equal or less than 1	Good
	More than 1	Unsuitable
Na%	< 20	Excellent
	20–40	Good
	40–60	Permissible
	> 60	Undesirable
*SAR*	< 10	Excellent
	10–18	Good
	18–26	Fair
	> 26	Unsuitable
*RSC*	< 1.25	Safe
	1.25–2.5	Marginal
	> 2.5	Unsuitable
*MR*	> 50	Suitable
	< 50	Unsuitable
*PI*	> 75%	Good — Class I
	25–5%	Good — Class II
	< 25%	Unsuitable — Class III

*KR* Kelly’s ratio, *Na%* sodium percent, *SAR* sodium absorption ratio, *RSC* residual sodium carbonate, *MR* magnesium ratio, *PI* permeability index

3. Sodium adsorption ratio

The SAR is a measurement of the sodium content or the alkali hazard and is used for estimating the suitability degree of water for irrigation purposes. The *SAR* ratio is calculated from the following equation:

$$SAR={ {\varvec{N}}{\varvec{a}}}^{+}/(\surd ({{\text{Ca}}}^{2+}+{{\text{Mg}}}^{2+})/2)\;{\text{meq}}/{\text{L}}.$$  

**Fig. 8:**
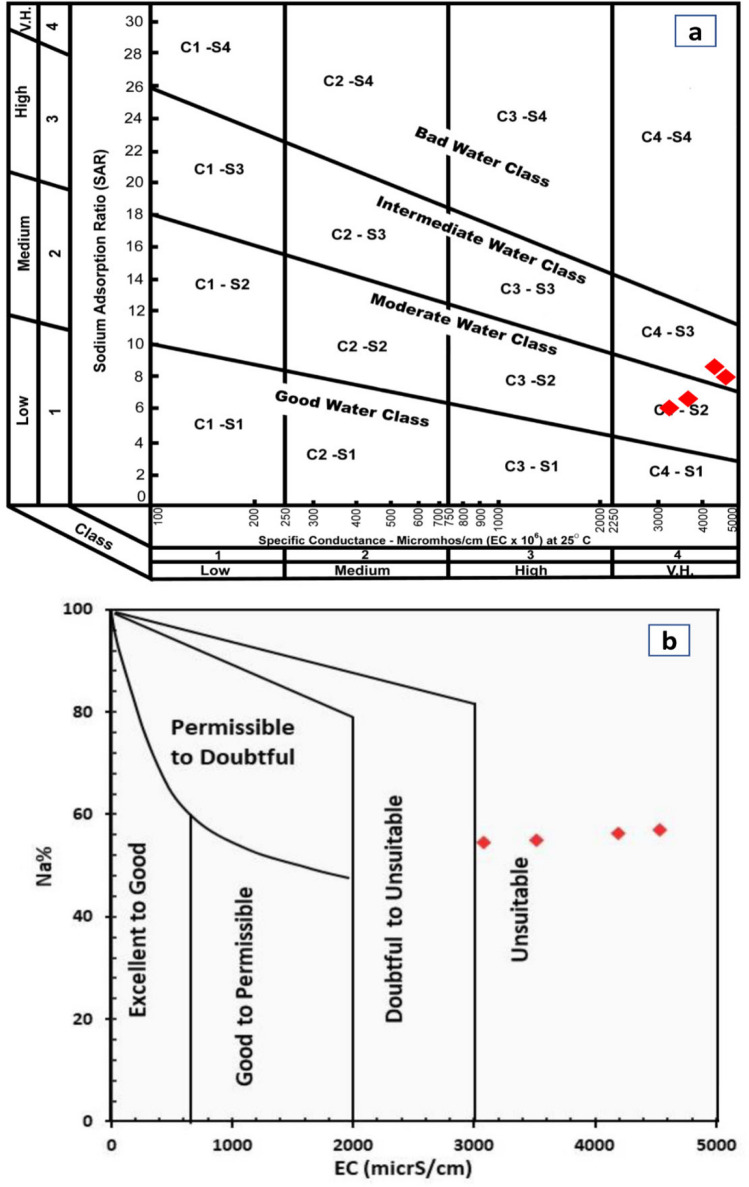
**a** US Salinity diagram and **b** Wilcox diagram for the classification of the studied water for irrigation purposes

The *SAR* ratio is an important parameter due to its direct relation to the sodium adsorption by soil (Rao 2006). Water quality classes based on *SAR* are given in Table 5. Based on *SAR*, all samples have *SAR* < 10 (Fig. 7c). Additionally, the samples were plotted on the US Salinity Laboratory staff (USSL Staff 1954) for the classification of water based on the coupled effect of salinity hazard (expressed by *EC*) and sodium hazard (expressed by *SAR*) indicated that the samples PP and PF plot in the C4-S2 field while samples PO and raw irrigation water plot in the C4-S3 field (Fig. 8a).

**Table 5:** Classes of USSL diagram (Zaman et al. 2018)

Salinity hazard	
C1	Used for irrigation of most crops on most soils with little development of soil salinity
C2	Used if a moderate infiltration can occur. Plants with moderate salt tolerance can be grown without special salinity control
C3	Cannot be used on soil with restricted drainage and poor infiltration; special salinity control may be required; and plants with good tolerance should be selected
C4	Not suitable for irrigation under ordinary conditions but may be used in special cases (soil must be permeable; drainage must be good; and irrigated water must be applied in excess to provide considerable infiltration). Only very salt tolerance plant should be selected
Sodium hazard	
S1	Used for irrigation under ordinary circumstances on all soil with little development of harmful levels of sodium content, whereas sensitive sodium crops as avocados and stone fruit trees may accumulate harmful concentrations of sodium
S2	It shows a remarkable sodium hazard in fine textured soils which have cation exchange capacity and low infiltration unless gypsum is applied in the soil. This water may be used in coarse textured soil or organic soils with good permeability
S3	It produces harmful sodium content in most soils. Its use requires good drainage, high infiltration, high organic conditions, and soil amendments. Chemical amendments may be not suitable for very high salinity waters
S4	It is unsatisfactory for irrigation uses except at low and perhaps medium salinity. Gypsum as soil amendment may facilitate the use of this class in irrigation

The US Salinity diagram divides the water into several classes C1, C2, C3, and C4 based on the salinity hazard, and S1, S2, S3, and S4 based on the sodium hazard. Zaman et al. (2018) discussed the properties and interpretation of each class (Table 5).

The application of Wilcox diagram (1948) indicates that the samples are unsuitable for irrigation based on Na% and *EC* (Fig. 8b).

4. Residual sodium carbonate

*RSC* is an empirical parameter for predicting the alkalinity hazard associated with CaCO_3_, MgCO_3_ (Eaton 1950). It is expressed as the following equation:

$$RSC= \left({{{\text{CO}}}_{3}}^{2-}+{{{\text{HCO}}}_{3}}^{-}\right)-\left({{\text{Ca}}}^{2+}+{{\text{Mg}}}^{2+}\right) {\text{meq}}/{\text{L}}.$$

Eaton (1950) and Wilcox *et al.* (1948) classified the water quality for irrigation purposes based on *RSC* into safe (< 1.25), marginal (1.25–.5), and unsuitable (> 2.5) (Table 4). The studied samples have *RSC* of < 1.25 (Fig. 7d).

5. Magnesium ratio

*MR* is used as a parameter to determine the suitability of water for irrigation, where *MR* greater than 50% indicates unsuitable (Paliwal 1972). This ratio is determined by the following equation:

$$MR=\left[\frac{{{\text{Mg}}}^{2+}}{{{\text{Ca}}}^{2+}+{{\text{Mg}}}^{2+}}\right]\times\;100\;{\text{meq}}/{\text{L}}.$$  

The presence of high Mg content in the soil reduces crop yield due to the resultant alkaline nature of soil (Kumar et al. 2007). According to *MR* classification, all samples have *MR* of less than 50% (Fig. 7e; Table 4).

6. Permeability index

The *PI* is used to measure the suitability of water for irrigation purposes when compared with the total ions in mmol l^−1^. This is due to the susceptibility of the soil permeability to be endangered because of the consistent use of irrigation water which increases the presence of sodium, calcium, magnesium, and bicarbonate in the soil (Chandu et al. 1995). The *PI* is expressed as follows:

$$PI=\frac{{{\text{Na}}}^{+}+\sqrt{{{\text{HCO}}}_{3}^{-}}}{{{\text{Ca}}}^{2+} +{\mathrm{ Mg}}^{2+} + {{\text{Na}}}^{+}}\times\;100\;{\text{mmol}}/{\text{L}}.$$  

According to the *PI*, all the studied samples represent water of good class for irrigation (Class I) (Fig. 7f; Table 4).

## Discussion

*Pleurotus ostereatus* (PO) performed best when grown on PDA and could also be grown on two synthetic media MDA (medium including infusion of *Salvia officinalis*) and TDA (medium including infusion of *Thymus vulgaris*) in a percentage of 70%, 69.11% compared with the standard PDA at 25 °C and pH 6.5. *Pleurotus floridanus* (PF) exhibited moderate growth on assayed natural media. It was grown also on MDA and TDA and recorded a percentage of growth, 65% and 55.82% from the corresponding slandered PDA. Finally, the least growth recorded by *Pleurotus pulmonarius* (PP) with percentage measured 62.24% and 43.34% on MDA and TDA, respectively, relative to PDA. The growth of all isolates shows no significant difference between their growth on either MDA or TDA with a significance decrease from standard PDA. The results also clarified a progressive reduction in fungal dry weight and its associated growth rates from standard PDA from MDA and TDA with mass growth parameters slightly exceed on MDA and TDA, among all tested *Pleurotu*s strains.

In a correlated study, Lee et al. (2015) suggested that the marked increase in mycelial growth through addition of *Salvia miltiorrhiza.* Fruiting body yields recorded highest mass yield measured at 139.5 g/850 mL in a substrate amended by 5 g/bottle of *Salvia miltiorrhiza*. Monika et al. (2020) also reported that the cultivation of *Pleurotus eryngii* on post‐extraction waste of *Thymus vulgaris* (TPEW) can improve beneficially the mushroom commercial yield by fluctuating mineral composition, phenolic compounds, antioxidant activity, and organic acids. In contrast during our study, the growth of mushroom on TDA gave the least radial growth with all tested *Pleurotu*s strains. PO, PF, and PP recorded 4.43, 3.31, 1.92, respectively, as shown in Table 1, and also the dry mats followed the same trend. This may be due to the inhibitory action of phenolic compounds that increased in fungal mats or due to the inhibitory action of the essential oil that is originally present in *Thymus* extracts. The essential oil of *Thymus* is a potent antibacterial and antifungal activity (Karaman et al. 2001). P-cymene, linalool, terpinene-4-ol, and thymol were the major inhibitory compounds (antimicrobial essential oils) of *T. vulgaris* (Bhaskara Reddy et al. 1998).

Heavy metals are found on the crust of earth in their regular form. They pose a hazard due to the bioaccumulation in the cells of living organisms (Baby et al. 2010). Continuous exposure to heavy metal causes deviation in normal human hygienic health and induces diseases like Parkinson’s disease, Alzheimer’s disease, multiple sclerosis, and muscular dystrophy; and chronic exposure to some heavy metals may be the cause of different types of cancers (Ghosh et al. 2007). Many filamentous white-rot fungi can remove pollutants especially heavy metals from groundwater in natural environment (Chen et al. 2011; Shivakumar et al. 2011; and Mishra and Malik 2012). The water remediation by microorganism is more efficient compared to physicochemical techniques, which are much costive, consume energy, and may induce secondary pollution (Price et al. 2001).

Fungi are able to neutralize the higher toxicity of the heavy metals through three main mechanisms including cell surface binding and intracellular and extracellular accumulation (Mishra and Malik 2012; Xu et al. 2014; Sazanova et al. 2015). Being metabolism independent, the adsorption on cell surface can occur in either viable or inactivated microorganisms, while the intracellular and extracellular accumulations of heavy metals are frequently energy-driven processes and occurred only in living cells (Sag 2001). Non-active microbial biomass usually exhibits a higher affinity for heavy metals compared with living biomass which commonly due to the lack of competing protons exhibits during their metabolism.

In the current work, there are a marked decrease in the concentration of most soluble heavy metals on the natural liquid medium (sterile drainage water/liquid PD broth) inoculated with three tested *Pleurotus* strains individually which indicated that the metals had been successfully removed from all fungal inoculated solutions, as shown in Table 3 and Fig. 5. At the end of the incubation period, *Pleurotus ostereatus* (PO) are more efficient in the removal of Al, Co, Cr, and Ni (53.15%, 95.87%, 58.47%, and 85.07%); respectively. *Pleurorotus pulmonarius* (PP), are more potent in the removal of Cd, Si, Sn, Sr, and V (70.37%, 56.59%, 41.19%, 52.78%, and 96.24%), respectively, while *Pleurorotus pulmonarius* (PP) is actively over the former species in the removal of Ba, Fe, Mo (87.84%, 46.67%, and 97.34%), respectively. While the variations among heavy elements (Cu, Mn, Pb, As, and Se) could not be detected in the control sample as well as in treatments, recorded measurements are below the instrumental detection.

Unexpected increase in Zn among the different treatments is from 05.04 to 07.01%; this may be attributed to the dissolution of Zn salts contaminated with potato extract by our tested organisms and hence the discharged Zn ions varied according to the selective potentiality of each *Pleurotus* strain. Hence, the slight increase in Zn ions were detected in all tested *Pleurotus* broth media. The removal of most heavy metals by *Pleurotus* strains suggests that the fungus was more tolerant to these elements. The absorbed metals from solution were chemically biotransformed by the fungus through two methods, intracellular accumulation and cell transport. The absorbed metals were extracellularly converted into insoluble metal compounds (Yang et al. 2017). Validating the higher toxicity of Cr and Cd over Pb is reported by Morcillo et al. (2015).

It was also evident that complexing with heavy metals and organic acids exhibited by some fungi depended separately on the efficiency of each metal species (Jarosz-Wilkolazka and Gadd 2003; Sazanova et al. 2015). It seems worthy to focus on the study carried out by Yang et al. (2017) which reported that all the concentrations of heavy metals decreased with gradual increase of the concentration of oxalic acid content in inoculated solutions, and some heavy metals were strongly chelated with oxalic acid to form complex compounds. This trail confirmed also that the newly formed insoluble metals included chelated and adsorbed metals.

The effect of heavy metals on fungal growth and viability appears to vary according to the concentrations and the kind of element. In general, low concentrations of several metals seem to stimulate fungal growth, while high concentrations inhibit growth due to induction of physical abnormalities, such as cellular membrane damage, stimulation of lipid peroxidation, and formation of reactive oxygen species, respiration suppression, modification of enzyme action, and molecular damage in DNA and proteins (Sazanova et al. 2015).

Milovanović et al. (2014) support that mycelial growth of *Pleurotus ostreatus* was good in media enriched with selenium as a heavy metal-supplement till 500 mg L^−1^, while 1000 mg L^−1^ was recorded as the Se-minimum inhibitory concentration for tested fungus. At Se concentrations up to 500.0 mg L^−1^, the mycelial characteristics were short, frequently septated and branched hyphae, with a highly intensive extracellular matrix, and lack of clamp connections. At high Se concentrations over 500 mg L^−1^, intact hyphae have no cellular contents, with high vacuolization levels, and with noticed numerous proteinaceous compositions. Besides *Pleurotus* spp., *Aspergillus lentulus* FJ172995 also has been shown to remove multiple heavy metals such as Pb, Cr, Cu, and Ni from their environment simultaneously by absorption as reported by Mishra and Malik (2012).

**Fig. 9:**
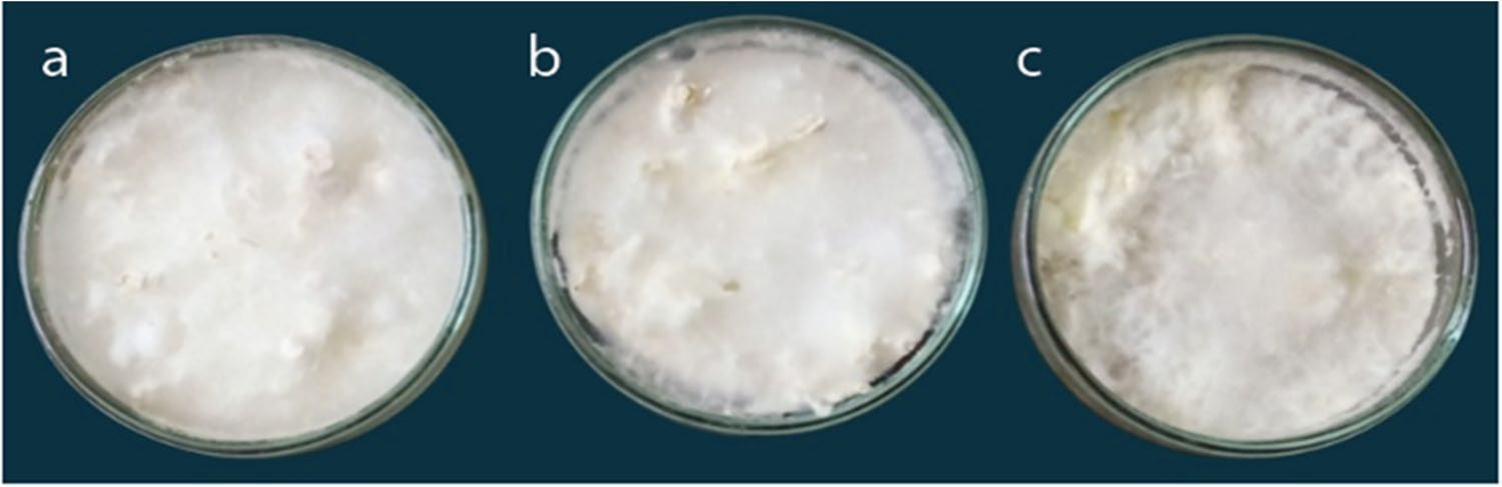
The mycelial growth of different *Pleurotus* spp. on sterile drainage water amended to the liquid PD broth (1:1 v/v): **a** P.O, **b** P.P, and **c** P.F

**Fig. 10:**
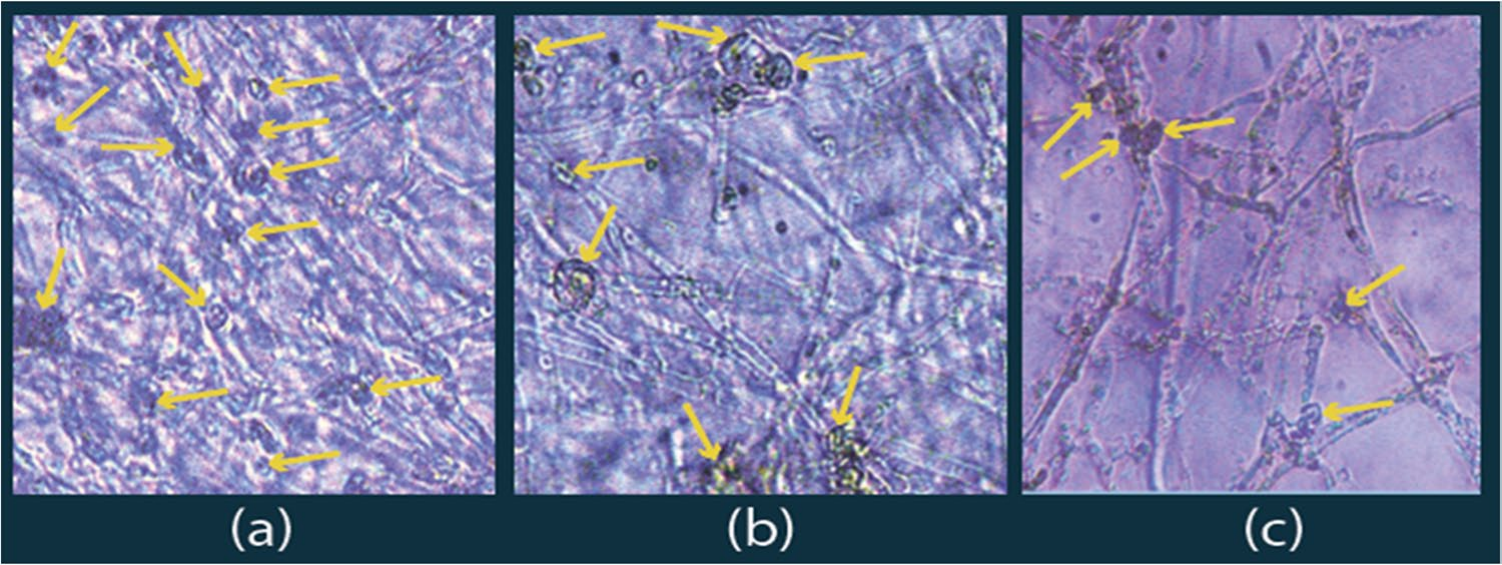
Hyphal morphology showing heavy metal accumulations in the different *Pleurotus* spp. grown on sterile drainage water amended to the liquid PD broth (1:1 v/v). **a** The yellow arrows represent hyphal vesicular formation among all tested isolates, **b** moderate compaction and vesicular formation (higher compaction and vesicular formation), and **c** less compaction and vesicular formation

In the current work, the microscopic examinations for hyphal extensions with three tested isolates are as shown in Figs. 9 and 10; these are displayed considerable formations of intercalary swallowing vesicular hyphal structures (high levels in PO, moderate in PP, and lower in PF).

**Fig. 11:**
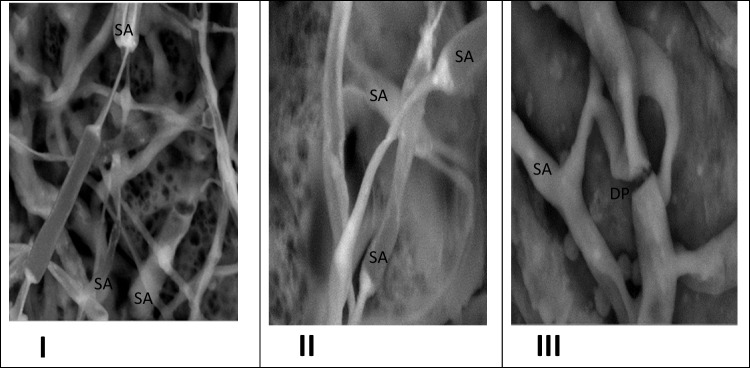
Hyphal morphology of *Pleurotus* spp. examined by a scan electron microscope (SEM), showing swollen areas (SA) in photos (I, II, and III) and a degradative portion (DP) only recorded with PF in photo no. (III). The *Pleurotus* spp. grown on sterile drainage water amended to the liquid PD broth (1:1 v/v), I (mycelial growth of PO), II (mycelial growth of PP), and III (mycelial growth of PF)

In this trend, Gharieb and Gadd (1998) found that vesicular mycelial structures may be attributed to high accumulation of heavy metals in hyphae followed by evaporation to less toxic organic forms through methylation pathway. Hyphal morphology of *Pleurotus* spp. examined also by a Scan Electron Microscope (SEM), showing swollen (SA) that may be exhibited due to accumulations of heavy metal and a degradative portion (DP) in mycelium of PF also may be created due to heavy metal stressors, as shown in Fig. 11.

The results of the water quality assessment of the raw irrigation wastewater and the three samples extracted after experiments showed that the suitability of this water for irrigation varies depending on the studied parameter. According to the *KR* parameter, the studied water samples are unsuitable for irrigation due to the increased Na content (Fig. 7a), while they show high Na% ranging from 54.4 to 57.1%, indicating permissible water quality for irrigation (Fig. 7b). Additionally, the *SAR* in all samples have *SAR* < 10 indicating excellent quality for irrigation uses (Fig. 7c). However, the use of US Salinity Laboratory staff (USSL Staff 1954) for the classification of water based on the coupled effect of salinity hazard (expressed by *EC*) and sodium hazard (expressed by *SAR*) indicated that the samples PP and PF plot in the C4-S2 field (very high salinity with medium sodium hazard), while samples PO and raw irrigation water plot in the C4-S3 field (very high salinity with high sodium hazard) (Fig. 8a). The studied samples have *RSC* of < 1.25 representing safe water for irrigation (Fig. 7d). *MR* in the studied samples is > 50%, indicating unsuitable water for irrigation. The presence of high Mg content in the soil reduces crop yield due to the resultant alkaline nature of soil (Kumar et al. 2007). *PI* shows that the studied samples show good class (Class I) (Fig. 7f).

From the above discussion of the suitability of water for irrigation, all the studied sample have salinity as well as sodium hazard, indicating that the irrigation waters should be applied when sufficient calcium from calcareous soils is dissolved to decrease the remarkable sodium hazard (Zaman et al. 2018; Nosair et al. 2022; and Patel et al. 2023). Additionally, these waters could be used in a permeable soil of good drainage with very salt tolerance plants. The presence of Na hazard favors the use of good drainage, high infiltration, high organic conditions, and the use of soil amendments. Chemical amendments may not be suitable for very high salinity waters (Paliwal 1972).

## Conclusions

Interdisciplinary work was done to help reduce the negative effects of global warming. The work targeted a new project in the western Egyptian Nile Delta (NED) to (1) effectively reuse agricultural wastewater in the cultivation of protective planting to the NED, (2) remediate the wastewater as effectively as to be used in the cultivation using three fungal species (*Pleurotus* spp.), (3) investigating the growth rates of the studied fungi on different substrates to get the optimum growth conditions, and (4) reduce stresses on the groundwater resource in the study area by using agricultural wastewater. The growth of *Pleurotus ostereatus* (PO) was the best when grown on PDA and could also be grown two natural media MDA and TDA in a percentage of 70% and 69.11% from the standard PDA. So, PO could be grown on recycled desert plant debris to reduce air pollution results from burning these wastes. PO is also effective in water remediation and could be employed as additional water treatments for further purification of drainage water compared to physicochemical techniques, which are much costive, consume energy, and may induce secondary pollution. PO could remove heavy metal in effective promising percentages (Co, 95.87%; Ba, 86.42%; Ni, 85.07%, Mo, 73.67%; Cr, 58.47%; Al, 53.15%; Cd, 36.11%; Si, 25.09%, Fe, 23.04%, Sr, 22.44%; V, 07.13%; and Sn, 02.42%). P.O could be ecofriendly used in new developing communities as NED for the remediation of drainage water to maximize its use as well as pose little stress on the groundwater resource.

The results of agricultural water quality assessment of the raw wastewater and the samples collected after the mycoremediation indicated that all samples have salinity and sodium hazard, indicating that the irrigation waters should be used when sufficient calcium from calcareous soils is dissolved to decrease the remarkable sodium hazard. The use of highly permeable soil of good drainage will be efficient in the cultivation process.

## Data Availability

The data used to support the findings of this study are available from the corresponding author upon request.
